# State of the Knowledge of VA Military Sexual Trauma Research

**DOI:** 10.1007/s11606-022-07580-8

**Published:** 2022-08-30

**Authors:** Tara E. Galovski, Amy E. Street, Suzannah Creech, Keren Lehavot, Ursula A. Kelly, Elizabeth M. Yano

**Affiliations:** 1 Women’s Health Sciences Division, National Center for PTSD, VA Boston Healthcare System, 150 South Huntington Street, Boston, MA 02130 USA; 2 Department of Psychiatry, Boston University School of Medicine, Boston, MA USA; 3 VA VISN 17 Center of Excellence for Research on Returning War Veterans, Central Texas VA, Waco, TX USA; 4 Department of Psychiatry, Dell Medical School, University of Texas at Austin, Austin, TX USA; 5 VA HSR&D Center of Innovation for Veteran-Centered and Value-Driven Care, VA Puget Sound Healthcare System, Seattle, WA USA; 6 Department of Psychiatry and Behavioral Sciences, University of Washington, Seattle, WA USA; 7 Department of Health Services, University of Washington, Seattle, WA USA; 8 Atlanta VA Health Care System, Atlanta, GA USA; 9 Nell Hodgson Woodruff School of Nursing, Emory University, Atlanta, GA USA; 10 VA HSR&D Center for the Study of Healthcare Innovation, Implementation & Policy, VA Greater Los Angeles Healthcare System, Los Angeles, CA USA; 11 Department of Health Policy & Management, UCLA Fielding School of Public Health, Los Angeles, CA USA; 12 Department of Medicine, UCLA Geffen School of Medicine, Los Angeles, CA USA

**Keywords:** military sexual trauma, literature review, research summary, veterans

## Abstract

Despite substantial efforts to counter sexual assault and harassment in the military, both remain persistent in the Armed Services. In February 2021, President Biden directed the U.S. Department of Defense to establish a 90-day Independent Review Commission on Sexual Assault in the Military (IRC) to assess the department’s efforts and make actionable recommendations. As servicemembers discharge from the military, effects of military sexual trauma (MST) are often seen in the Veterans Health Administration (VA). In response to an IRC inquiry about VA MST research, we organized an overview on prevalence, adverse consequences, and evidence-based treatments targeting the sequelae of MST. Women are significantly more likely to experience MST than their male counterparts. Other groups with low societal and institutional power (e.g., lower rank) are also at increased risk. Although not all MST survivors experience long-term adverse consequences, for many, they can be significant, chronic, and enduring and span mental and physical health outcomes, as well as cumulative impairments in functioning. Adverse consequences of MST come with commonalities shared with sexual trauma in other settings (e.g., interpersonal betrayal, victim-blaming) as well as unique aspects of the military context, where experiences of interpersonal betrayal may be compounded by perceptions of institutional betrayal (e.g., fear of reprisal or ostracism, having to work/live alongside a perpetrator). MST’s most common mental health impact is posttraumatic stress disorder, which rarely occurs in isolation, and may coincide with major depression, anxiety, eating disorders, substance use disorders, and increased suicidality. Physical health impacts include greater chronic disease burden (e.g., hypertension), and impaired reproductive health and sexual functioning. Advances in treatment include evidence-based psychotherapies and novel approaches relying on mind-body interventions and peer support. Nonetheless, much work is needed to enhance detection, access, care, and support or even the best interventions will not be effective.

## INTRODUCTION

Despite substantial efforts to counter sexual assault and harassment in the military, both remain persistent in the Armed Services. In February 2021, President Biden directed the U.S. Department of Defense to establish a 90-day Independent Review Commission on Sexual Assault in the Military (IRC) to assess the department’s efforts and make actionable recommendations. The IRC work was organized around four lines of effort: accountability, prevention, climate and culture, and victim care and support. Given VA’s research infrastructure, leaders of the fourth line (care and support) requested a briefing on VA military sexual trauma (MST) research. This paper provides the resulting overview on the definition and prevalence of MST, then broadly describes the range of adverse consequences associated with MST, including mental and physical health outcomes, functioning, and well-being. We then describe the research on evidence-based care targeting negative outcomes, with a focus on posttraumatic stress disorder (PTSD). This paper is not intended to be an exhaustive review of all studies conducted in the VA on MST; instead, it is a broad overview of the state of the knowledge on this important topic. The preponderance of included studies was conducted by VA researchers. We also included several non-VA studies to enhance knowledge throughout.

### Defining MST

MST is the term used by the VA to refer to experiences of sexual assault or sexual harassment during a period of military service. The specific definition of MST, from U.S. Code 1720D, is “physical assault of a sexual nature, battery of a sexual nature, or sexual harassment (unsolicited verbal or physical contact of a sexual nature which is threatening in character) which occurred while the former member of the Armed Forces was serving on duty, regardless of duty status or line of duty determination.” As such, any experience of sexual assault or harassment while serving in the U.S. Armed Forces regardless of the reason for the assault, whether the assault occurred on or off base, or whether the assault was perpetrated by another member of the military, is included in the VA’s definition of MST. The VA definition of MST is distinct from other definitions of sexual trauma in its inclusion of sexual harassment, defined as “unsolicited verbal or physical contact of a sexual nature which is threatening in character.”

### Prevalence of MST

It is difficult to determine the exact prevalence of MST as methodological differences across studies contribute to a range of estimates. Data from self-report surveys and interviews typically result in higher prevalence compared to estimates derived from VA medical records.^1^ Estimations of MST in VA recruited samples tend to be higher than in non-VA and mixed samples.^2^ The prevalence of MST among men has been understudied relative to women and, as a result, is particularly difficult to estimate.^3^ A recent meta-analysis conducted with studies of military personnel and Veterans suggested that approximately 16% report MST with marked gender differences, such that proportionally more women (38%) than men (4%) reported experiences of MST.^2^ When sexual assault and harassment were considered individually, experiences of harassment were more common, with 31% of military personnel and Veterans experiencing harassment (52% of women and 9% of men) and 14% sexual assault (24% of women and 2% of men).

Beyond women’s elevated risk for MST, administrative data suggest that other groups with low societal and institutional power (e.g., young age, low education, unmarried, lower rank, more recently entered service, short time in the duty unit) are also at increased risk of experiencing MST.^4^ When compared to non-LGBT servicemembers who reported MST (14%), servicemembers who identified as sexual minorities (26%) and as transgender (30%) were more likely to report MST.^5^

### Adverse Consequences of MST

Notably, not all MST survivors experience long-term adverse consequences or require clinical care. However, for a substantial number of MST survivors, the adverse consequences of MST can be significant, chronic, and enduring. Research conducted in VA has focused primarily on VA users and has revealed that the complexity of the aftermath of MST is apparent across a host of co-occurring mental and physical health outcomes as well as in the cumulative impairments in functioning across major life domains. The impact of MST varies widely across survivors, and the cumulative effect of MST can be significantly influenced by known social determinants of health. Given the universe of potential adverse outcomes of MST, there is no clear formula for assessing the impact of MST and no single solution for intervening in its effects.

Sexual trauma that takes place within military settings shares many commonalities with sexual trauma that occurs elsewhere. Sexual victimization often represents significant interpersonal betrayal that can disrupt existing views of the world, self, and others. Survivors of sexual trauma may encounter “rape myths” that blame the victim for the assault or justify the perpetrator’s actions (e.g., “It wasn’t rape if he was drinking”). For male survivors of sexual trauma, gender-specific rape myths may increase male gender role stress^6^ which, when related to suppressing emotionality in male military personnel, was associated with more severe PTSD symptoms.^7^ To try to make sense of the event, survivors may attempt to identify *why* this happened and may come to faulty conclusions about their responsibility for the assault (e.g., “I could have prevented this. This is my fault”), resulting in significant experiences of guilt and shame.

To fully understand the adverse consequences of MST, the unique personal, social, political, and occupational context of this type of trauma must also be acknowledged. Institutional betrayal can compound interpersonal betrayal. Those who experience MST frequently report that the military institution contributed to an environment in which MST was common, likely to occur, and difficult to report.^8–10^ Many MST survivors report fear of reprisal if they speak openly of their experiences, including the possibility of additional violence, demotions, and unwanted job reassignments.^11,12^ Other feared consequences include ostracism by colleagues, isolation from peers, loss of support, and disruptions in unit cohesion.^13^ Concerns about disruptions in unit cohesion and fears for the implications of MST on military careers may be more salient for male Veterans.^3^ Survivors of MST may experience subsequent distrust of both comrades and command.^10^ The disintegration of the support structure in the military is uniquely egregious not only because the nature of the job requires trusting one another with one’s very life, but also because military service includes working with *and* living with one’s fellow soldiers.^14^ Having to continue to work and live side by side with one’s perpetrator, and potentially rely on one’s perpetrator for safety in life-threatening situations, presents an environment from which escaping the effects of MST may be impossible. This context compounds distress and complicates later help-seeking.^10,15–17^

#### Mental Health Impacts

PTSD is the most common psychiatric condition to develop secondary to MST.^18^ The risk for developing PTSD for women Veterans who experienced MST is well documented. When compared to women Veterans without a history of MST, women Veterans who experienced MST were 9 times more likely to develop PTSD.^13,19^ Moreover, MST is more strongly associated with PTSD among women Veterans relative to their male counterparts.^20^ While the risk for PTSD may be higher for women who experience MST, research has also shown that PTSD symptoms may be more severe for men who have experienced MST than for women.^21^ Taken together, studies demonstrating elevated risk for developing PTSD secondary to MST suggest that the unique aspects of military exposure (e.g., stigma, reporting barriers, institutional betrayal) may amplify distress and increase the likelihood of diagnosable mental illness.^19,22^

Notably, PTSD rarely occurs in isolation. Women Veterans who report MST and have PTSD are more likely to receive a host of comorbid mental health diagnoses, including major depression, anxiety, eating disorders, and substance use disorders.^23,24^ Indeed, MST is associated with increased odds of receiving any mental health diagnoses.^18^ Experiences of MST also exacerbate pre-existing mental health conditions and increase the severity of mental health disorders including PTSD, depression, substance use disorders, eating disorders, and insomnia.^18,25–29^

The scope and severity of mental health sequelae following MST include the ultimate negative outcome—suicide. Even after controlling for other risk factors, MST independently predicts suicidal ideation and attempts.^16^ Notably, the majority of Veterans who reported post-MST suicidality experienced the onset of suicidal ideation (68%) and attempted suicide for the first time (75%) *after* MST.^16^ MST is an independent risk factor for suicide mortality among Veterans using VA services, including MST services.^30^ The integration of cross-cutting prevention initiatives within MST care and suicide prevention at VA is critical to identifying risk and intervening to prevent suicides.

#### Physical Health Impacts

Similar to mental health outcomes associated with MST, physical health consequences are broad and variable across individuals. MST history in women is associated with a host of chronic medical illnesses including increased risk for diabetes mellitus, hypertension, obesity, and cardiovascular risk factors.^31^ In a sample of women Veterans who served in the Gulf War, MST was linked to gastrointestinal, genitourinary, musculoskeletal, and neurological symptoms.^32^ In a study of women Veterans seeking treatment in the VA for PTSD related to MST, two-thirds reported chronic pain.^33^ Physical health consequences of MST can be further complicated by the negative mental health consequences described earlier. Comorbid mental health disorders can complicate receipt of medical care, furthering amplifying the negative effects on physical health.^34^

The impact of MST on reproductive health and sexual functioning in women Veterans is well-established including a higher likelihood of having a sexual dysfunction disorder, sexually transmitted infections, infertility, perinatal depression, sexual pain, and reporting low sexual satisfaction.^35–38^ Difficulties in sexual functioning secondary to MST were significantly more severe in women Veterans than that which was associated with childhood sexual abuse alone.^35,36^ In a recent study, women Veterans who reported MST were less likely to have a full-term birth infant, more likely to have a lower birthweight infant, and more likely to report experiencing postpartum depression and/or anxiety above and beyond age at pregnancy, racial/ethnic minority status, childhood violence exposure, and warfare exposure.^39^

#### Functional Impairment

The impact of the experience of MST on survivors’ functioning can be immediate, and the sequelae can be enduring. In a study of women Veterans deployed to post 9/11 wars, the experience of MST negatively impacted reintegration efforts including relationships with family, and occupational and educational functioning.^40^ Military sexual harassment specifically was linked with impairment in post-military functioning across major life domains including satisfaction with employment for women and satisfaction with romantic relationships and parenting for both women and men.^41^ Post-deployment homelessness has also been associated with MST, particularly in male Veterans.^42^ Impairments in functioning can persist over time and have significant implications for long-term quality of life. For example, in a recent study of Vietnam-era women Veterans, military sexual harassment and discrimination conferred significant risk for continued impairment for over 40 years in later life functioning and increased rates of disability, even after accounting for psychological and psychiatric distress.^43^ Military sexual harassment was also associated with decreased physical functioning, also an important indicator of quality of life and successful aging.^43^

### Intervening in the Sequelae of MST

#### Screening

While primary prevention of MST research falls largely outside of the domain of the VA, screening, detection, and secondary prevention efforts are critical parts of the VA response to MST. Like sexual trauma that occurs outside of military settings,^44^ most experiences of MST go undetected by formal helping systems.^45,46^ Many MST survivors may be reluctant to disclose their trauma history to healthcare providers, either due to expectations of or prior experiences with negative, invalidating and blaming reactions to disclosure^8,47^ or, perhaps, due to fear of posttraumatic stress responses that describing traumatic events can elicit.^48^ The Veterans Health Administration (VHA) implemented a universal MST screening program^49^ designed to aid providers in identifying Veterans who have experienced MST and to facilitate their access to no-cost treatment for MST–related health conditions. Analysis of medical record data demonstrates that VHA’s MST screening program is feasible and yields clinically meaningful information, and that identification of a history of MST through screening increases the likelihood of mental health treatment.^19,50^ Importantly, VA patients have described their experiences with disclosures to VA providers to be generally positive, although a subset of male Veterans, regardless of MST history status, reported lower satisfaction.^51^ That said, male Veterans are less likely to access psychiatric care for MST–related distress than women Veterans or may delay care longer.^52^ In summary, despite the barriers to care for all MST survivors noted in qualitative studies, quantitative research revealed that Veterans with a positive MST screen were more likely to engage in healthcare in VA.

#### Assessment

Once a patient reports the experience of MST, the next step is to assess the extent and scope of the sequelae of this experience with respect to mental and physical health outcomes and impacts on functioning. Sexual violence is explicitly noted in the *Diagnostic and Statistical Manual of Mental Disorders*, 5^th^ edition, as an example of a Criterion A traumatic event, a necessary but not sufficient condition for a diagnosis of PTSD.^53^ Some MST experiences that involved sexual harassment victimization in the absence of sexual assault victimization will reach the threshold for a Criterion A traumatic event, while others will not.^54^ While harassment-only MST may result in PTSD and other mental health conditions, sexual assault MST shows a stronger association with a host of negative outcomes including more severe PTSD, depression, sexual functioning, and suicidal ideation.^55^ The results of a comprehensive assessment of the MST event and associated symptoms will best inform the potential diagnoses and personalized intervention. As noted, the heterogeneity of potential adverse consequences of MST is substantial and interventions for each outcome are beyond the scope of this paper. We focus on an overview of VA’s evidence-based care for PTSD given that this is the most common mental health condition associated with MST.

#### Treatment for MST–Related PTSD

Systematic reviews and meta-analyses, summarizing the efficacy of interventions by evaluating multiple studies, reveal that psychotherapy for PTSD yields larger effects in treating PTSD than pharmacotherapy.^56^ Overall, trauma-focused treatments are more effective than non-trauma-focused treatments.^57^ Based on these studies and independent reviews of randomized, clinical trials, clinical practice guidelines (CPGs) have been independently developed by workgroups from internationally recognized professional organizations and healthcare systems, including a VA/DoD workgroup (VA/DoD, 2017). Decisions about the strength of the evidence for an existing intervention are based on the empirical evidence for that intervention. The quality of the evidence for effectiveness of Prolonged Exposure^58^ (PE) and Cognitive Processing Therapy^59^ (CPT) has been given the highest ratings across CPGs.

Both PE and CPT were originally developed in the civilian population and initially tested with female survivors of sexual trauma. The VA has been a leader in continuing to develop, evaluate, and implement PE and CPT within the Veteran population, including randomized clinical trials (RCTs) including patients with PTSD secondary to MST. Indeed, because these interventions were originally developed for PTSD due to sexual trauma, the translation to target the sequelae of MST was not a leap.^60–63^ In addition to RCTs, effectiveness studies testing these therapies in real-world clinical settings have demonstrated the effectiveness of these evidence-based practices (EBPs) in both non-residential and residential VA services.^64–66^ Given the effectiveness of these interventions, the VA has dedicated substantial resources to the historic, large-scale dissemination effort of these therapies via the Mental Health Dissemination Initiative. As a result, PE and CPT have been widely disseminated across VA resulting in access to the gold standards of care for PTSD for Veterans enrolled in the healthcare system.^67,68^

Despite the evidence base supporting the effectiveness of PE and CPT, there is room for improvement. Among RCTs in active duty and Veteran populations, PTSD symptom effect sizes from pre- to posttreatment range from *d* = 0.78 to 1.02 and 35–40% of participants fail to experience clinically meaningful improvement.^69,70^ Premature attrition from treatment for all trauma survivors suffering from PTSD is also problematic. For example, at least 35% of all Veterans who initiate a course of CPT in VA clinics fail to complete therapy, resulting in suboptimal doses of treatment which contributes to poor outcomes.^71^ Initiating treatment and continued engagement in treatment are difficult, and MST survivors may be faced with various barriers to care including grappling with institutional betrayal, experiencing stigma about mental health needs, and not recognizing symptoms as PTSD^10,42,72^ as well as gender-related barriers.^52^ Despite these barriers to engaging in care for PTSD that may be unique to MST, post 9/11 Veterans were more likely to initiate^73^ and complete treatment^74^ for PTSD as compared to Veterans who did not experience MST. Likewise, attrition rates from residential VA care for PTSD did not differ between those who experienced MST and those patients who did not.^75^ In the same study, those who reported MST experienced larger treatment gains but did not maintain those gains to the extent that non-MST patients did after discharge. PE and CPT are examples of trauma-focused interventions which, by definition, ask the patients to engage with the trauma memory that they have been working very hard to avoid—in other words, to break through the avoidance that has been keeping them trapped in PTSD and preventing recovery. Thus, the very avoidance of the trauma memory (a hallmark symptom of PTSD) may be prohibitive for engaging in the treatment for the disorder.^76^

There is emerging evidence that non-trauma-focused therapies (nTFT) may also be effective in reducing PTSD symptoms^60^; however, fewer studies on nTFTs have been conducted specifically focusing on treating PTSD secondary to MST, with some exceptions. Examples of nTFTs include Present-Centered Therapy^63^ (PCT), which is currently recommended by several CPGs, and Interpersonal Therapy^77,78^ (IPT) and Stress Inoculation Therapy^79^ (SIT), which are recommended only by the VA/DoD CPG. Given that the evidence is not as strong for nTFTs as it is for trauma-focused treatments, these interventions are recommended for patients who are not able to engage in trauma-focused treatments for any number of reasons.^80^ Skills Training in Affective and Interpersonal Regulation^81^ (STAIR) is a preparatory treatment designed to target affective dysregulation and interpersonal difficulties that may prevent patients from engaging in trauma-focused therapy, and one CPG issued a standard recommendation for its use.

Finally, there is emerging evidence to support complementary, integrative, and alternative treatments in VA. A systematic review of mind-body interventions, such as mindfulness, yoga, and relaxation techniques, indicated promise in reducing PTSD symptoms.^82^ Kelly and colleagues^83^ recently published interim results from an ongoing trial comparing trauma-sensitive yoga to group CPT for women who were diagnosed with PTSD secondary to MST. The results indicated that both interventions were effective in achieving sustained, clinically meaningful improvements and that retention rates were higher in the yoga therapy condition.

Strategies designed to support and supplement evidence-based care are emerging. Peer support is one such strategy. Designed to target major risk factors for poor outcomes after trauma including loneliness, thwarted belongingness, institutional betrayal, and isolation,^84^ peer support may mitigate these effects independently as well as offer a conduit to clinical care and support during an MST survivor’s journey through recovery.^85^ The Women Veterans Network (WoVeN) is an example of a national peer support network with a developed 8-week core curriculum designed to increase connection and support for women Veterans.^86^

Certainly, many MST survivors do not access VA care and/or do not have access to evidence-based community care. While the experience of MST is associated with VA healthcare utilization, this association may be mediated by mental health needs (e.g., PTSD or depression).^87^ Some survivors of MST may not need or want mental health treatment or may prefer to receive care in a different setting. Alternative strategies might provide educational and interventional resources for those who are not engaged in services within or outside of VA. Mobile and web-based mental health strategies may serve the function of reducing barriers to care, increasing access, and increasing choice for Veterans at all stages of recovery. Delivery of Self Training and Education to Stressful Situations-Women Veterans (DESTRESS-WV) is an example of an online, coach-assisted intervention that was specifically tailored for women Veterans with PTSD and with demonstrable efficacy in reducing PTSD.^88^ Beyond MST is a free, secure, and private self-help mobile app developed by VA specifically to support the health and well-being of MST survivors. The app has specialized psychoeducation, assessments, and skills-based tools to help Veterans who experienced MST cope with challenges, manage symptoms, improve their quality of life, and find hope.^89,90^

## CONCLUSION

The VA has funded MST research for over 25 years, resulting in significant gains in our knowledge base through efforts from groups such as the National Center for PTSD, Women’s Mental Health, the national MST Support Team, and the VA Women’s Health Research Network. In July 2021, the IRC released their final report with 82 recommendations for their four priority areas: accountability, prevention, climate and culture, and victim care and support.^91^ The recommendations for victim care and support were informed by the type of VA studies highlighted in this overview and include suggestions for optimizing the victim services workforce within the Department of Defense, expanding victim service options to meet the needs of all sexual harassment and assault survivors, centering survivors to facilitate healing and restorative justice, and re-envisioning training and research to improve victim care and support.

Despite great strides, this work is far from complete. Areas for further research include continued inquiry into MST survivors’ service needs and continued improvement in screening and detection of those needs; the impact of MST on health behaviors and overall physical health consequences including gynecologic care, and fertility; and the risk for subsequent trauma. There is also a need for research documenting the intergenerational impact of MST on Veteran family functioning including parenting, parent-infant bonding, and risk for adverse outcomes among dependents. Continued study with Veterans who experienced MST but do not use the VA is necessary, as their health status is largely unknown. Research should continue to explicate the role of social determinants of health on both access to care and effectiveness of care, particularly for racial, ethnic, and sexual minority Veterans, with consideration of important intersections between systemic racism, sexism and heterosexism with trauma, healthcare, and military service.

The adverse consequences of MST are complex and include mental and physical negative outcomes as well as significant impairment in functioning across major life domains. The patterns of outcomes are variable across survivors of this type of trauma, and no single intervention can be universally applied for recovery. The complexity of clinical presentations requires personalized approaches to care that leverage recent advances in health technology. Flexible interventions that may vary significantly across survivors will best accommodate the wide range of psychiatric disorders and physical health complications, and address the impact on functioning. Finally, barriers to reporting MST and receiving care must be removed or even the best of interventions will not be effective. In conclusion, further research in organizational, secondary prevention, and intervention science will continue to guide important policy decisions in preventing and effectively responding to sexual trauma during military service.
